# Influenza vaccination coverage rates in five European countries during season 2006/07 and trends over six consecutive seasons

**DOI:** 10.1186/1471-2458-8-272

**Published:** 2008-08-01

**Authors:** Patricia R Blank, Matthias Schwenkglenks, Thomas D Szucs

**Affiliations:** 1 Institute of Social- and Preventive Medicine, University of Zurich, Hirschengraben 84, 8001 Zurich, Switzerland; 2 European Center of Pharmaceutical Medicine, University of Basel, Blumenrain 23, 4051 Basel, Switzerland

## Abstract

**Background:**

The objectives of the survey were to identify the level of influenza vaccination coverage in five European countries between 2001 and 2007, to understand the drivers and barriers to vaccination, to assess vaccination intentions for the winter 2007/08 as well as major encouraging factors for vaccination.

**Methods:**

Between 2001 and 2007, representative household surveys were performed with telephone or mailed (France) interviews of individuals aged 14 and above. The questionnaire used in the UK, Germany, Italy, France and Spain was essentially the same in all seasons. The data were subsequently pooled. Four target groups were defined for the analysis: 1) persons aged 65 years and over; 2) persons working in the medical field; 3) chronically ill persons and 4) combined target group composed of individuals belonging to one or more of the previous groups 1, 2 or 3.

**Results:**

In 2006/07, vaccination coverage was, 25.0% in UK, 27.4% in Germany, 21.8% in Spain, 24.2% in France and 24.4% in Italy. During six influenza seasons (2001–2007), vaccination coverage showed a slight positive trend in the five countries (p ≤ 0.0001). In the elderly (≥ 65 years), across all countries, no significant trend was seen; the vaccination rate decreased non-significantly from a peak of 64.2% in season 2005/06 to 61.1% in season 2006/07. The most frequent reason for getting vaccinated was a recommendation by the family doctor or nurse (51%), and this was also perceived as the major encouraging factor for vaccination (61%). The main reason for not getting vaccinated was feeling unlikely to catch the flu (36%).

**Conclusion:**

In the UK, Germany and Spain, influenza vaccination coverage rates in season 2006/07 dropped slightly compared to the previous season. However, a trend of increasing vaccination coverage was observed from 2001/02 to 2006/07 across Europe. The family doctor is the major source of encouragement for individuals getting vaccinated. Efforts to overcome the barriers to vaccination need to be put in place to reach the WHO objective of 75% coverage in the elderly by 2010. This is a major challenge to be faced by governments, healthcare workers and healthcare organisations.

## Background

Seasonal influenza is one of the principal causes of vaccine-preventable disease with up to 500,000 deaths per year worldwide [1]. It can cause mild to severe illness, whereas the very young, the elderly and those with certain chronic health conditions are at risk for serious influenza complication [2,3]. Influenza is a contagious respiratory illness caused by influenza viruses. Point mutations occur during viral replication and lead to frequent antigenic changes. Antigenic drift results in new influenza virus variants which can cause seasonal Influenza epidemics and require the modification of the vaccine each year [4]. However, the primary strategy for preventing the flu is getting vaccinated against influenza annually. The surveillance network for humans and targeted influenza vaccination programmes including specific contingency plans, adequate stockpiling of antivirals, investing in pandemic vaccine research and promoting domestic production of influenza vaccines are cornerstones in national and international pandemic preparedness plans [5].

Several studies evaluated the economic impact of vaccination, particularly among children, healthy working adults and the elderly. Cost savings were most evident in children under high risk conditions by diminishing indirect costs caused by parental working days lost due to the care of their sick children [6]. The vaccination of healthy adults has shown to be cost-effective and cost saving by reducing the indirect costs which are associated with work absenteeism or affected work productivity [7]. The elderly are the group at highest risk for influenza complications. Hence, vaccination of this part of the population provides considerable economic benefits [8].

In the five European countries covered by this survey, official vaccination recommendations are very alike. They cover: 1) people aged 65 years and over (as an exception, Germany uses a cut-off of 60 years), 2) individuals of age 6 months or above with high risk chronic conditions including cardio-pulmonary diseases, diabetes mellitus and immunologic disorders, or HIV/AIDS (except in Italy where the latter group is not included), 3) nursing home residents, 4) health and social care workers [9,10]. Some countries make additional recommendations which were not explicitly covered by this survey [11,12]. The age-based recommendations for 60+ and 65+ respectively have been implemented before 2001/02 and were not changed during the whole study period.

Despite public awareness and efforts by policy makers, physicians and health care providers, influenza vaccination rates and vaccine manufacturing capacities need to be increased substantially to achieve the degree of baseline preparedness required in case of a surge in immunization uptake when a influenza pandemic occurs.

There is evidence in earlier publications investigating vaccination coverage rates in Europe that the threat of avian influenza impacted on vaccination rates in several European countries [13-15]. This survey, based on cross-sectional data, was conducted in order to examine changes in influenza vaccination coverage during six consecutive seasons and to monitor behaviours, with a particular focus on high risk target groups. Furthermore, related motivations and barriers, and vaccination intentions for the coming season 2007/08 were addressed.

## Methods

This survey is an ongoing international assessment of influenza immunization uptake in Germany, UK, Italy, France and Spain. New countries added in 2006/07 were Austria, Czech Republic, Finland, Ireland, Poland and Portugal. However, this report only covers the original five countries. During six influenza seasons, from 2001/02 to 2006/07, population-based surveys were carried out in December and January among households representative of the non- institutionalized population. The same methodology was used from the beginning. Except in France, interviews were realized via computer-assisted telephone interviews (CATIs) conducted by TNS healthcare (formerly Département Santé TNS Sofres). The method of the fieldwork was described earlier [16]. The overall response rate was 52.6% (UK 33%, Germany 29%, Italy 77%, Spain 57%, France 67%). Data from Spain were available since 2002/03. French data were collected via a postal questionnaire by GEIG (Groupe d'Etude et d'Information sur la Grippe). 3991 people aged 15 years and over responded to the questionnaire. In order to adjust the sample size with that of the other countries, French data were weighted according to standard criteria to represent 2000 individuals instead of 3991. The survey population was representative of the adult population aged 14 years or older in Germany, Italy and Spain, 15 years or older in France and 16 years or older in the UK. In Spain, individuals over 75 years of age we not included in the survey.

According to the Esomar World Research Codes and Guidelines this type of study is considered market research and does not require the approval of an ethics committee, as this survey is a research in people, which are deemed healthy and not in the medical system [9]. At the beginning of each call, the agreement and explicit verbal consent of the interviewees was obtained. There was no study intervention and the anonymity of the participants was guaranteed. To obtain a representative sample of the national non-institutionalized population aged 14 and over, interviews were carried out according to quotas and a weighting was applied in terms of gender, age, profession, geographic region and town size. Quotas and weighting factors were based on data from official national sources [17].

Four target groups based upon international recommendations were determined as follows [18-22]:

1. Individuals aged 65 years or older

2. Individuals who suffer from a chronic illness

3. Individuals working in the medical field (health care workers)

4. Composite target group (individuals aged 65 and over or who suffer from a chronic illness or who are healthcare workers)

Individuals belonging to none of the four above-defined target groups were classified as members of the non-target group (seasons 2003/04–2006/07).

According to official recommendations of the five countries' governments, the group of chronically ill is defined as children, adolescences and adults suffering from chronic diseases of respiratory organs, including asthma, chronic heart disease, chronic renal disease, diabetes, congenital or acquired immune deficiencies) [2,6,8,9,23-25].

The survey questions have been published earlier [17]. The questionnaire covered vaccination uptake, reasons for and against vaccination, as well as general opinions towards influenza vaccination. After 2003/04, additional information on chronic illnesses was collected. In order to identify chronically ill persons, examples were provided to the respondents. Questions regarding influenza pandemics and avian influenza were added from season 2005/06.

The annual datasets were pooled (for analysis across all five countries) and sample weights were applied to correct for small deviations from the age and gender quotas requested. SPSS^® ^version 14 for Windows was used for the statistical evaluation. Bivariate association of categorical variables were assessed with the Chi squared test and the Chi squared test for trend was used for evaluating time trends of these variables. For all statistical tests, two-sided p = 0.05 was used as the level of statistical significance. If available, exact p-values were displayed. Ninety-five percent confidence intervals (CI) were reported as appropriate. Covariates identified as potential predictors of influenza vaccination in univariate analysis were considered as candidates for multivariable analysis. Logistic regression was used to identify independent correlates of the outcome of interest, i.e. vaccination coverage. Due to the descriptive nature of this data, no correction for multiple testing was made.

## Results

### Demographic data

During season 2006/07, 186,285 telephone contacts were built up and 6,000 mails sent out. The questionnaire was completed by 12,036 individuals. The overall sample consisted of 58,027 interviewed persons since 2001. Table 1 gives an overview of the sample for the year 2006/07 only. The samples were similarly composed over the years and were representative of the population aged 14 or older [17]. Spain was appeared to have a smaller proportion of persons older than 65 years, as the survey did not cover persons over 75 years of age in this country.

**Table 1:** Overview of sample in season 2006/07

	**UK**	**Germany**	**Italy**	**France**	**Spain**	**All**
**Total **(N)	2037	2007	2001	2000	2000	**10045**
**Mean age **(years)	45	47.6	45.1	46.4	42	**45.2**
(95% CI)	(44.1; 45.8)	(46.8; 48.4)	(44.3; 45.9)	(45.6; 47.2)	(41.3; 42.7)	**(44.9; 45.6)**
**Female**	51.4%	51.9%	51.3%	51.9%	50.4%	**51.4%**
(95% CI)	(49.4%; 54.4%)	(49.9%; 53.9%)	(49.3%; 53.3%)	(49.9%; 53.9%)	(48.4%; 53.4%)	**(50.4%; 52.4%)**
**Age ≥ 65 years**	18.9%	24.0%	17.4%	20.0%	12.3%	**18.5%**
(95% CI)	(17.9%; 20.9%)	(22.0%; 26.0%)	(16.4%; 19.4%)	(18.0%; 22.0%)	(11.3%; 14.3%)	**(18.7%; 19.1%)**
**Work in the medical field**	6.4%	8.5%	4.2%	7.0%	4.9%	**6.2%**
(95% CI)	(5.3%; 7.4%)	(7.3%; 9.7%)	(3.3%; 5.1%)	(5.8%; 8.1%)	(4.0%; 5.8%)	**(5.7%; 6.7%)**
**Chronic illness**	14.5%	24.6%	13.9%	16.9%	13.2%	**16.6%**
(95% CI)	(12.9%; 16.0%)	(22.7%; 26.5%)	(12.4%; 15.4%)	(15.3%; 18.6%)	(11.7%; 14.6%)	**(15.9%; 17.3%)**
**Combined target group**^**1**^	33.2%	44.5%	29.4%	36.1%	25.7%	**33.8%**
(95% CI)	(31.1%; 35.2%)	(42.3%; 46.7%)	(27.4%; 31.4%)	(34.0%; 38.2%)	(23.8%; 27.6%)	**(32.9%; 34.7%)**

^1 ^Includes people aged 65 years or over, suffering of chronic illness or working in medical field

### Vaccination rates in the general population

**Figure 1:**
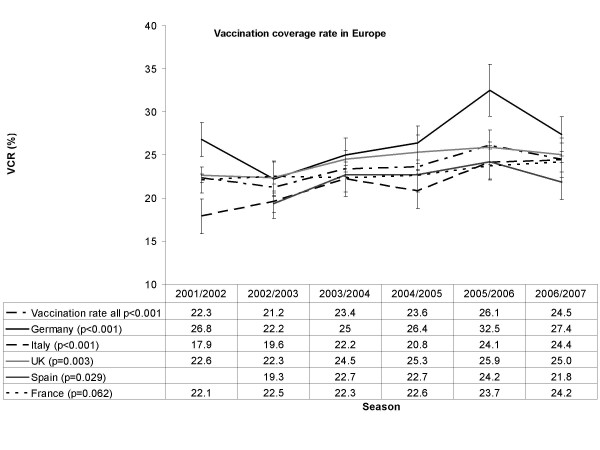
Seasonal Influenza Vaccine Coverage in the general population in 5 European countries by season (2001/02–2006/07).

Overall vaccination coverage rates across all surveyed countries are shown in Figure 1. The decreased rate in season 2006/07, compared to season 2005/06, was statistically significant (p = 0.012). It was mainly due to coverage changes in Germany and Spain. The rates seen in the other countries were essentially constant. In contrast, across all six seasons, a significant positive trend (p ≤ 0.0001) was identified overall and in the UK, Germany, Italy and Spain. The differences in coverage between the five countries were statistically significant (p = 0.002).

**Figure 2:**
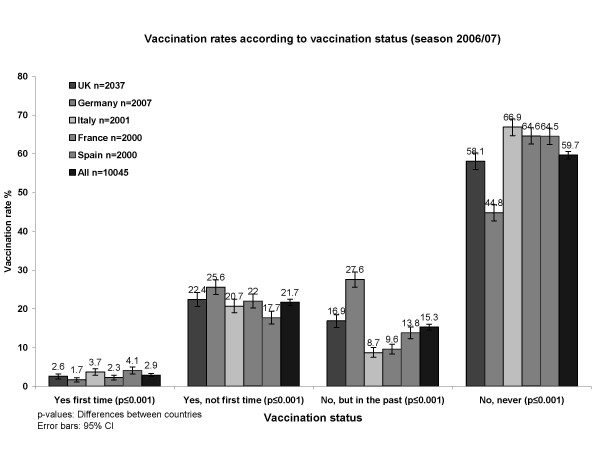
Vaccination rates according to vaccination status (season 2006/07).

Vaccination status in season 2006/07 is shown in Figure 2. Across all six seasons a statistically significant decrease in the overall proportion of never vaccinated individuals was evident (chi-squared test for trend, p ≤ 0.0001). However, the overall proportion of individuals vaccinated in the past but not in season 2006/07 increased by 2.0%. The overall proportion of first time immunizations was significantly lower by 4.0% in 2006/07 compared to 2005/06 (p ≤ 0.0001).

With regard to the coming winter of 2007/08, across all countries, 36.6% of the interviewees intended to get vaccinated against the flu (-2.4% compared to the year before). In all years and across all countries, vaccination intentions were higher than the actual coverage rates achieved. The ratio between actual and intended vaccination coverage (across countries) remained stable over the years (range: 0.63 in 2001/02 and 0.7 in 2004/05). The gaps were highest in Germany and lowest in France.

With respect to gender, no significant associations were found at the country level in season 2006/07. In the UK, a slightly stronger vaccination attendance among females was detected (unadjusted OR 1.2, p = 0.062), whereas in Spain men were more likely to get an immunisation (unadjusted OR: 0.9, p = 0.123). Across all six seasons, a significant gender difference was noted in the UK (p = 0.0001, men<female), in Italy (p = 0.004, men>female) and in Spain (p = 0.005, men>female).

### Vaccination rates and trends in target groups

Predictors of vaccination (age, working in the medical field and suffering from chronic illness) were investigated using multivariate logistic regression. The reference group were individuals who belonged to the non-target group. The effect of gender was statistically not relevant and was debarred from the final multivariate analysis.

#### Age

**Figure 3:**
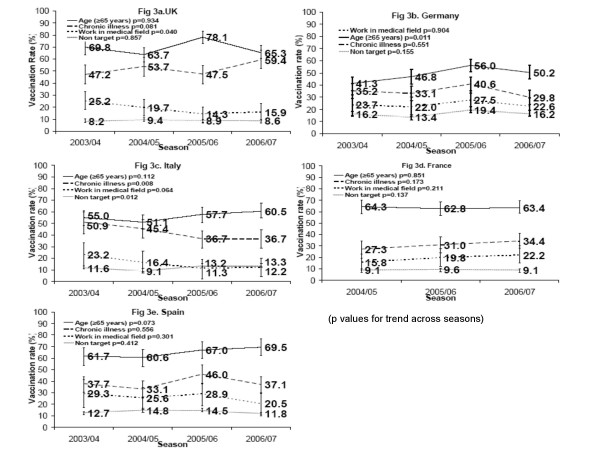
**Trend curves of actual vaccination rates in high-risk target groups (vs. non-target group) and in the non-target group in all five countries (seasons 2003/04–2006/07).** 3a. UK. 3b. Germany. 3c. Italy. 3d. France. 3e. Spain.

**Figure 4:**
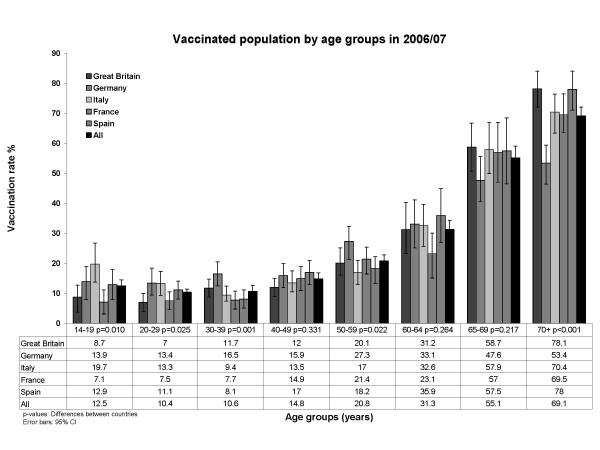
Vaccinated population by age group (season 2006/07).

Figures 3 show that the highest coverage among the elderly above age 65 was achieved in Spain (69.5%, 95% CI: 62.5%; 76.5%) (Fig 3e). Germany had the lowest coverage rate (50.2%; 95% CI: 44.2%; 56.2%) even though the only statistically significant time trend of increasing coverage rates (p = 0.011) was found here (Fig 3b). Vaccination coverage in season 2006/07 by age group is shown in Figure 4.

Table 2 shows the multivariate-adjusted impact of belonging to the elderly target group, on the probability of getting vaccinated, which was similar in seasons 2006/07 and 2005/06. Since 2003/04, an overall upward trend could be statistically confirmed (p = 0.005).

**Table 2:** Adjusted odds ratios of vaccination coverage in target groups vs. the non-target group (adjustment for age ≥ 65 years, chronic illness, working in the medical field), in season 2006/07

	**UK**	**Germany**	**Italy**	**France**	**Spain**	**All**
	(n = 2026°)	(n = 1996°)	(n = 2001)	(n = 3798°)	(n = 1994°)	(n = 11,815°)
Age*	20	5.2	9.9	17.4	16.9	11.9
(95% CI)	(14.6; 27.3)	(3.9; 7.0)	(7.3; 13.4)	(12.6; 24.0)	(11.7; 24.3)	(10.3; 13.6)
p-value	<= 0.0001	<= 0.0001	<= 0.0001	<= 0.0001	<= 0.0001	<= 0.0001
N	258	234	260	759	168	1679
						
Chronic illness*	15.5	2.2	3.8	5.3	4.4	4.8
(95% CI)	(10.8; 22.2)	(1.6; 3.0)	(2.7; 5.5)	(3.7; 7.5)	(3.1; 6.3)	(4.1; 5.5)
p-value	<= 0.0001	<= 0.0001	<= 0.0001	<= 0.0001	<= 0.0001	<= 0.0001
N	186	231	159	321	172	1069
						
Chronic illness and age*	51.9	7.2	22.2	49.7	21	20.1
(95% CI)	(30.1; 89.6)	(5.2; 10.0)	(13.9; 35.5)	(29.4; 84.1)	(12.4; 35.8)	(16.6; 24.4)
p-value	<= 0.0001	<= 0.0001	<= 0.0001	<= 0.0001	<= 0.0001	<= 0.0001
N	119	167	125	354	78	843
						
Work in medical field *	2	1.5	0.9	2.9	1.9	1.8
(95% CI)	(1.2; 3.5)	(1.0; 2.4)	(0.4; 1.8)	(1.8; 4.7)	(1.1; 3.3)	(1.4; 2.3)
p-value	0.012	0.065	0.772	<= 0.0001	0.026	<= 0.0001
N	118	122	73	222	84	619
						
Work in medical field and chronic ill or age*	3.9	1.1	5.4	7.4	6.2	3.2
(95% CI)	(1.4; 10.9)	(0.5; 2.5)	(1.6; 18.3)	(2.6; 21.1)	(2.2; 17.1)	(2.1; 5.0)
p-value	0.01	0.749	0.007	<= 0.0001	<= 0.0001	<= 0.0001
N	17	39	11	30	16	113

°N < total sample size for this season due to missing covariate values.

*Reference category: non-target group (persons who do not belong to any target group)

#### Chronic illness

A question concerning the vaccination status of chronically ill persons was added to the questionnaire in 2003/04. In the presence of chronic illness (like heart or lung disease, diabetes or others), vaccination rates were higher than in the non-target group. In season 2006/07, they ranged from 29.8% (95%CI 23.8%; 35.8%) in Germany to 59.4% (95% CI 52.4%; 57.4%) in the UK. (see Figs 3).

Correspondingly, chronic illness was a strong predictor of vaccination (Tab 2). Being old and chronically ill boosted the adjusted ORs in all five countries even more. They reached 20.1 (95% CI 16.6; 24.4) on average. In France, the probability of getting vaccinated was nearly ten times higher than in the group of chronically ill persons who were not old.

#### Working in the medical field

In season 2006/07, as in the previous seasons, there were substantial differences between the individual countries: in Germany, the rate of vaccinated medical care workers (22.6%, CI 14.6%; 29.6%) was nearly twice as high as in Italy (12.2%, CI 4.2%; 20.2%) (Figs 3). Over time, healthcare workers showed a continuous decrease in immunization uptake compared to the non target group. Only France showed an increasing but not significant trend (p = 0.217).

It is consistent with these findings that working as a health care professional was not a strong driver of vaccination in multivariate regression (Tab 2).

#### Combined target group and non-target group

In the composite target group (including individuals aged 65 and over or who suffered from a chronic illness or who were healthcare workers) a vaccination rate below 50% was only seen in Germany (40.9%, CI 37.9%; 43.9% in 2006/07). Spain (50.6%, CI: 45.6%; 54.6%), Italy (51.0%, CI: 47.0%; 55.0%), France (51.4%, CI: 48.4%; 55.4%) and UK (57.9%, CI: 53.9%; 61.9%) showed a higher coverage by far. Vaccination coverage in the non-target group was essentially stable across seasons (Figs 3).

### Drivers and barriers to vaccination

Table 3 shows reasons for getting and not getting vaccinated in season 2006/07. The principal driver of vaccination was receiving an advice from the family doctor (average 51%, previous season 49%). Media attention on avian flu led 6% of the overall population to get a flu immunisation (previous season 7%). In France, the most frequently stated reason was, like in the previous year, that the vaccine is paid by the social security system. Rankings of these driving forces did not change to a large extent.

**Table 3:** Ranking of reasons for and against vaccination in season 2006/07 (First six ranks)

**Motivations for getting vaccinated**	**UK**	**Germany**	**Italy**	**France**	**Spain**	**All**
	(n = 509)	(n = 549)	(n = 488)	(n = 485)	(n = 435)	(n = 2465)
	Rank (%)	Rank (%)	Rank (%)	Rank (%)	Rank (%)	Rank (%)
My family doctor/nurse advised me to do it	1 (60)	2 (71)	1 (43)	3 (34)	1 (44)	1 (51)
Because the flu is a serious illness and I did not want to get it	2 (50)	1 (90)	2 (24)	3 (34)	2 (37)	2 (48)
Because of my age	3 (42)	5 (39)	5 (15)	2 (37)	3 (30)	3 (33)
Because I am not in very good health	4 (32)	6 (31)	3 (23)	6 (15)	4 (21)	6 (25)
So I do not pass the flu bug to my family/friends	5 (32)	3 (69)	4 (17)	5 (17)	5 (19)	5 (32)
Because the social security system pays for it	6 (29)	4 (46)	6 (6)	1 (47)	6 (19)	4 (30)
	
**Reasons for not getting vaccinated (among those never vaccinated)**	**UK**	**Germany**	**Italy**	**France**	**Spain**	**All**
	(n = 1183)	(n = 900)	(n = 1338)	(n = 1291)	(n = 1289)	(n = 6001)
	Rank (%)	Rank (%)	Rank (%)	Rank (%)	Rank (%)	Rank (%)

I do not think I am very likely to catch the flu	3 (33)	1 (48)	2 (22)	3 (18)	1 (63)	1 (36)
I have never considered it before	2 (35)	6 (31)	1 (38)	2 (21)	2 (43)	2 (34)
My family doctor did not recommend it to me	1 (38)	4 (39)	4 (14)	4 (17)	3 (25)	3 (26)
I am too young to be vaccinated	4 (32)		5 (12)	1 (35)	4 (18)	4 (22)
It is not a serious enough illness		3 (40)	3 (17)		6 (11)	5 (18)
I don't like injections/needles				6 (11)	5 (13)	6 (14)
My doctor at work has never recommended it to me	5 (21)					
My pharmacist has never recommended it to me	6 (21)	5 (33)				
I thought about it but I didn't end up getting vaccinated		2 (47)				
I don't think the vaccine is effective enough			5 (12)			
It is too expensive/not reimbursed				5 (17)		

With respect to barriers to vaccination, some differences were observed between the five countries covered (Tab 3). The answers given were in accordance with the respective data for 2005/06.

Knowledge about influenza and vaccination was very similar as in season 2005/06. Across all five countries, frequent statements were "you can catch the flu even if you are vaccinated" (65.0%) or "the infection is less sever if you are previously vaccinated" (54.9%). It is noteworthy that 22.6% were in agreement with the opinion that one will not catch the flu if one is vaccinated and that the vaccine will protect oneself in case of avian influenza or an influenza pandemic (18.6%). Do not know answers were below 32% for all items.

Answers regarding encouragements to vaccination are shown in Table 4. For France, only a limited amount of data was available concerning this topic.

**Table 4:** Encouragements for vaccination in season 2006/07

	**UK**	**Germany**	**Italy**	**France**	**Spain**	**All**
	(n = 2037)	(n = 2007)	(n = 2001)	(n = 2000)	(n = 2000)	(n = 10045)
	Rank (%)	Rank (%)	Rank (%)	Rank (%)	Rank (%)	Rank (%)
If my family doctor/nurse recommended it to me	1 (72)	1 (68)	1 (55)	1 (37)	1 (73)	1 (61)
If I had more information on the vaccine regarding efficacy and/or tolerance	2 (46)	2 (55)	2 (30)	-	2 (37)	2 (34)
If I knew more about the disease	4 (37)	3 (52)	3 (18)	-	3 (29)	3 (27)
If I could be vaccinated at work	6 (30)	5 (42)	8 (9)	3 (12)	3 (29)	4 (24)
If there were other ways of administering the vaccine (orally, injection without needle)	5 (34)	6 (40)	5 (11)	4 (10)	5 (24)	5 (24)
If it where cheaper/reimbursed/free	7 (25)	4 (44)	6 (10)	2 (24)	8 (7)	6 (22)
If my pharmacist recommended it to me	3 (39)	7 (33)	7 (9)	6 (3)	6 (17)	7 (20)
If there was more information on it generally	-	-	-	5 (9)	-	-
I would not change my mind	8 (9)	8 (10)	4 (17)	-	7 (11)	8 (10)

- No data available

## Discussion

Every year, the burden of seasonal flu is considerable. Vaccination coverage among non- and high-risk target groups is still not sufficient although effectiveness of flu vaccination is clearly demonstrated. To improve vaccination uptake, it is indispensable to gain insight into the vaccination uptake among different risk groups and to deal with the drivers and barriers to vaccination, in the population.

During six consecutive seasons (2001–2007), national surveys in the UK, Germany, Italy, France and Spain have been conducted to examine influenza vaccination coverage rates. Monitoring of the coverage in different target groups were carried out by telephone interviews using a CATI system (except in France), which is known as a suitable way of establishing influenza vaccination coverage in different groups of the population [26,27]. Mobile only users were not integrated in the survey as they are usually hard to reach and the costs of the interviews would be boosted. In France, self-administered questionnaires were sent out by letter. This methodology was already used successfully in the past [28,29].

During the season 2006/07, the vaccination coverage rate in the total sample decreased slightly to 24.5% compared to the previous season. However, a general trend towards slightly increasing coverage rates in the 5 European countries covered may still be intact.

Vaccination coverage in the elderly, in chronically ill persons and in persons working in the medical field varied strongly across countries and was rather low in some cases. A high proportion of vaccinated non-target group members in Germany (defined as persons <65 years, not chronically ill, not working as health care workers) can be attributed to the fact that here, other than in the remaining countries, vaccination is recommended for people aged 60 and over [30].

In the UK and Germany, 2006/07 compliance rates with influenza vaccination recommendations in those aged 65 years or older were below the level of 2005/06. In contrast, Italy, Spain and also France indicated higher vaccination rates in their elderly populations. Kroneman et al. found similar coverage rates in the elderly populations of Spain (67%) and Germany (53%) [31]. All five countries met the WHO objective of realising a 50% coverage in the elderly population in 2006, although Germany showed a borderline situation in this respect.

Another predisposing factor favourably affecting vaccination rates are chronic illnesses. A decrease in coverage, with respect to this target group, was seen in Spain and Germany. High coverage in UK may have been due to the fact that general practitioners were encouraged to proactively recommend the vaccine to their at risk patients [22]. The observation of a larger proportion of chronically ill persons in Germany compared to the rest of the observed countries remained unclear, since the wording of the question was the same in all five countries. Interestingly, this result is in line with an earlier cross-sectional study, which found a 20 percent proportion of Germans who stated to belong to this group [31].

The fact of suboptimal influenza immunization rates in health care professionals is well known [32]. Our study indicated a coverage which was lower than in all previous seasons covered by this survey. A low activity level of the influenza virus and a related late start of the influenza seasons may have reduced the attention of healthcare professionals in the last two years [33,34]. On the other hand, a survey using anonymous questionnaires carried out at the University Hospital Frankfurt, Germany, indicated a distinctly higher vaccination rate of 35.8%, while still disclosing a low acceptance of influenza vaccination among the healthcare professionals [35]. In single centres with a defined strategy, it may be possible to achieve noticeably higher vaccination rates than in all health care workers on average (including those who are not institution-based). This may provide an explanation for this apparent discrepancy. Interestingly, healthcare workers do not only regard the vaccination as a protection for their patients, but in the first instance as a protection for themselves [36].

Our study showed that the most important encouraging factors for reaching higher coverage rates were a proactive behaviour of healthcare workers as well as the implementation of effective public communication and education campaigns on influenza and influenza vaccine. Perception of the flu as a serious illness and recommendation by a family doctor were the principal reasons for getting vaccinated. In the literature, the advice of healthcare professionals is constantly reported to have a positive influence on coverage rates [15,37,38]. The attention on avian flu was not a strong driving force in season 2006/07. Fear of catching the bird flu may have waned due to reduced media coverage of influenza and avian influenza [39]. Hence, delayed vaccination campaigns due to late availability of the vaccine and decreasing public attention on flu and avian flu as a result of reduced media coverage and weakening political attention may have contributed to the decrease of immunisation uptake seen in some countries in season 2006/07.

Considering the entire study population, the ratio of actual and intended vaccination rate was 0.67 across Europe. Only France seemed to have a more realistic view, if they will be vaccinated the coming winter or not (actual/intended vaccination rate = 0.84). However, the discrepancy between vaccination intentions and actually receiving vaccination shows the potential for an increase in vaccination rates in the future. Efforts should be made at national and international levels to enhance the coverage rates and to achieve the WHO objectives of a 75% coverage in 2010 in the elderly population.

## Conclusion

In Europe, influenza vaccination coverage needs to be improved. By encouraging health care workers to proactively propose the influenza vaccination to the at-risk groups and to get their own flu shot, by wide and effective public communication and education campaigns on influenza as a disease and the mode of action of the vaccine, and by adequate funding, vaccination coverage rates can be increased. In five European countries covered here, coverage in the elderly exceeds the goal of the WHO and the European Parliament (The European Parliament resolution [P6_TA(2005)0406] on the strategy against an influenza pandemic, 26 October 2005) to vaccinate 50% of the aged population by 2006. However, efforts have to be made at the national and international levels to enhance the coverage rates in all target groups to achieve the WHO objective of a 75% coverage in the elderly in 2010.

## Competing interests

This study was made possible by an educational grant from the European Vaccine Manufacturers Group of the European Federation of Pharmaceutical Industries and Associations (EFPIA), Brussels, Belgium.

## Authors' contributions

PRB performed the data analysis, contributed to the data interpretation and wrote the manuscript. MS contributed to data analysis, data interpretation and the final version of the manuscript. TDS designed the project, contributed to the data acquisition and the analysis and supervised its development. All authors have read and approved the final manuscript.

## Pre-publication history

The pre-publication history for this paper can be accessed here:


